# A Prognostic Symptom Model Incorporating Patient-Reported Symptoms for Transplant-Ineligible Patients with Multiple Myeloma

**DOI:** 10.3390/cancers17030489

**Published:** 2025-02-01

**Authors:** Amaris K. Balitsky, Rinku Sutradhar, Hsien Seow, Anastasia Gayowsky, Alissa Visram, Jason Tay, Irwindeep Sandhu, Hira Mian

**Affiliations:** 1 Department of Oncology, McMaster University, Hamilton, ON L8V 5C2, Canada; balitsky@hhsc.ca (A.K.B.);; 2 Hamilton Health Sciences, Juravinski Cancer Center, Hamilton, ON L8V 5C2, Canada; 3 Escarpment Cancer Research Institute, Hamilton, ON L8V 5C2, Canada; 4 Institute for Health Policy, Management and Evaluation, University of Toronto, Toronto, ON M5T 3M6, Canada; 5 Division of Biostatistics, Dalla Lana School of Public Health, University of Toronto, Toronto, ON M5T 3M7, Canada; 6 ICES McMaster, Hamilton, ON L8S 4K1, Canada; 7 Department of Medicine, University of Caalgary, Calgary, AB T2N 1N4, Canada; 8 Department of Oncology, University of Alberta, Edmonton, AB T6G 2G3, Canada

**Keywords:** multiple myeloma, patient-reported outcomes, symptoms

## Abstract

Introduction: Patients with transplant-ineligible (TIE) multiple myeloma (MM) have high rates of symptom burden. The aim of this study was to develop and validate a prognostic model to predict symptoms in patients with TIE MM. Methods: In this population-based, retrospective cohort study, using multiple administrative health care databases linked using a unique encrypted patient identifier in Ontario, Canada, symptoms were identified using the patient self-reported Edmonton Symptom Assessment System (ESAS) at each clinic visit. The primary outcome was the presence of moderate-to-severe (ESAS score 4–10) symptoms (specifically symptoms of pain, tiredness, depression, and impaired well-being) within one year from the index date. Using the entire cohort, a multivariable logistic regression model with baseline covariates was developed to predict the risk of experiencing each of the above symptoms, categorized as moderate to severe within 1 year post-index date. Internal validation of the model was assessed via bootstrap validation methods. Results: A total of 1535 TIE adults with MM met the inclusion criteria. The median age was 75, with 25.2% of patients aged 80 years or older. In the multivariate analysis, baseline symptoms continued to be most associated with future symptom burden. Baseline severe pain (OR 9.84, 95% CI 6.29–15.7) was most associated with patients experiencing moderate–severe pain one year post-index date. Similarly, baseline severe tiredness (OR 17.34, 95% CI 9.00–33.42), baseline severe depression (OR 28.07, 95% CI 15.96–49.38), and baseline severely impaired well-being (OR 4.12, 95% CI 2.30–7.37) were the biggest predictors of patients experiencing moderate–severe tiredness, depression, and impaired well-being, respectively, at one year after the index date. Conclusions: Patients with MM experience persisting symptoms of pain, tiredness, depression, and impaired well-being, with baseline symptoms being the biggest predictor of future symptom burden.

## Simple Summary

Patients with transplant-ineligible multiple myeloma have high rates of symptom burden. We developed a tool to predict symptoms in this patient population using large datasets. Using symptoms reported by patients, we could predict who will experience persisting symptoms of pain, tiredness, depression, and impaired well-being. This tool can help identify patients who are high-risk and may benefit from psychosocial or symptom control interventions.

## 1. Introduction

Multiple myeloma (MM), a cancer characterized by the clonal proliferation of malignant plasma cells, is associated with major morbidity and mortality. The median age at diagnosis is 70 years, and so the majority of patients with MM are older and transplant-ineligible (TIE) at diagnosis [1]. Although the outcomes for TIE patients have improved over time, they still lag behind those in younger, transplant-eligible patients, with high rates of early mortality and symptom burden [2,3].

A number of prognostic tools have been developed in MM, including the international myeloma staging system (ISS), the revised international myeloma staging system (R-ISS), and additional stratification tools that incorporate more complex genetic and molecular markers [4,5,6,7]. While these tools are helpful for a clinician to use to provide the patient prognostic information about survival, they are not patient-centered, as they do not predict patient-reported outcomes (PROs) such as quality of life or patient-reported symptoms. PROs reflect the patient’s day-to-day experiences and patient burden with treatment.

Monitoring PROs as an intervention in cancer care offers substantial benefits, enhancing the overall quality of treatment and patient outcomes [8,9]. Studies have shown that the systematic tracking of symptoms allows for the earlier detection of complications and more timely interventions, which can significantly improve patients’ quality of life and improve treatment compliance and survival rates [10,11]. PROs play a crucial role in patient decision making, extending beyond the traditional focus on survival benefits, so patients can make decisions that align more closely with their personal values and preferences. This is especially important among older adults with MM, where over 75% of the patients prioritize quality of life, including functional and cognitive independence, over outcomes such as overall survival [12].

In this study, we aimed to develop and validate a prognostic model to specifically predict future symptoms in patients with TIE MM. The objective of this prognostic model is to provide patients with TIE MM prognostic information on their symptoms, which may allow them to engage in further conversation regarding their future needs and goals with their health care team. Once patients and clinicians can predict future symptoms, they can start to develop strategies to improve or prevent those symptoms.

## 2. Methods

### 2.1. Study Design and Population

We performed a population-based, retrospective cohort study. Multiple administrative health care databases in the universal, single-payer, publicly funded system in Ontario, Canada, were linked using a unique encrypted patient identifier and analyzed at ICES (formerly known as the Institute for Clinical Evaluative Sciences). The use of the data in this project is authorized under section 45 of Ontario’s Personal Health Information Protection Act (PHIPA) and does not require review by a Research Ethics Board.

All adults (age ≥ 18) with a new diagnosis of MM (International Classification of Diseases for Oncology, 3rd Edition, histology code 9732) between January 2007 and December 2018 were identified. In the Canadian landscape, the decision to transplant is made at the time of diagnosis due to the different subsequent publicly funded treatment pathways for transplant-eligible and TIE patients, respectively. However, as there are no specific diagnostic codes that would allow us to distinguish between transplant-eligible and -ineligible patients, TIE MM patients were defined as patients who did not receive a transplant within one year of MM (index date) diagnosis and were alive at the 1-year mark. This 1-year mark following MM diagnosis was defined as the index date for the prediction model development rather than the date of diagnosis to ensure we were capturing patients who had not undergone a transplant as first-line treatment.

We used the Edmonton Symptom Assessment System (ESAS) to identify patients with TIE MM who had completed patient-reported symptoms (at least one symptom score for pain, tiredness, depression, and overall well-being completed in the six months prior to the index date). In the event of multiple reported scores in the preceding 6 months, the worst score was considered the baseline. The ESAS Database started in 2007 when Cancer Care Ontario mandated the systematic screening of outpatients with cancer for symptoms using the ESAS. The ESAS is a validated and reliable patient-reported outcome tool that is used to assess nine common cancer-associated symptoms, including pain, tiredness, depression, well-being, drowsiness, nausea, appetite, shortness of breath, and anxiety [13]. Patients report the severity of each symptom from 0 to 10, with moderate symptoms defined as those with scores 4–6 and severe symptoms defined as those scoring 7 or higher [14]. Independent scores for each symptom are reported for clinicians to visualize.

### 2.2. Treatment Exposures

The treatment algorithm for the management of TIE MM is largely uniform in Canada’s universal health care system [15]. Given the evolving landscape of drug reimbursement, the standard-of-care regimens for front-line treatment of TIE MM changed during the study time-frame; treatment with fixed-duration proteasome inhibitor (PI)-based regimens like bortezomib–melphalan–dexamethasone (VMP) or cyclophosphamide–bortezomib–dexamethasone (CyBORd) for a total of 8–12 cycles has been funded since 2009, lenalidomide–dexamethasone (Rd) was first reimbursed in 2017, lenalidomide–bortezomib–dexamethasone (RVd) has been available since 2020, and daratumumab–lenalidomide–dexamethasone (DRd) has been the standard-of-care reimbursed regimen since 2022. Therefore, due to limited data availability, patients treated with RVd and DRd were excluded from this study.

### 2.3. Data Sources

We used multiple linked administrative databases (see Supplementary Table S1): Patient co-morbidities were defined using previous health care encounters including outpatient clinic visits and hospital discharge summaries. Patient self-reported symptoms were captured using the ESAS Database, as described above. The patient-reported functional score (PRFS), which is a patient-reported version of the palliative performance scale (PPS), was used to report patient performance status [16]. The ECOG performance status is comparable to and highly correlated with the PPS [17,18].

### 2.4. Outcomes

The primary outcome was the presence of moderate-to-severe (ESAS score 4–10) symptoms within one year following the index date. We derived models for the three most prevalent symptoms including pain, tiredness, and impaired well-being, as shown in our previous work [19]. Additionally, we included the symptom depression to capture a non-physical symptom.

### 2.5. Covariates

A separate prediction model was derived for each of the 4 symptoms described above. Each model included the following baseline covariates: age, sex, distance from cancer center (>50 km or more), co-morbidities (within 5 prior years), previous history of cancer in the last 15 years, low blood counts (hemoglobin < 100 g/L, platelets < 100 × 10^9^/L, and neutrophils < 1.0 × 10^9^/L), radiation, immunomodulatory drug/proteasome inhibitor (IMID/PI) drug usage, hospitalization or emergency department visit (≥1 occurrence), functional status, and baseline symptom burden (defined as each single symptom with the highest score). Distance from cancer center can capture geographic marginalization and a potential variable in predicting barriers to care and therefore symptom control. Performance status and symptom burden were the two patient-reported outcomes included among the covariates. Unless otherwise indicated, all covariates were measured within six months prior to the index date.

### 2.6. Statistical Analysis

#### 2.6.1. Prediction Model Development

Using the entire cohort, for each symptom, a multivariable logistic regression model with baseline covariates was developed to predict the risk of experiencing a moderate-to-severe symptom within 1 year post-index date. To build a parsimonious model, a backwards stepwise selection procedure with a liberal 2-sided *p* value of <0.10 was used to retain variables. Missing data from variables were handled by creating a missing data category.

#### 2.6.2. Prediction Model Validation

Due to the limited sample size, internal validation of the final developed model was assessed via bootstrap validation methods. This consisted of drawing patients randomly (with replacement) from the entire cohort until we reached the same sample size as the original cohort. This process was repeated 500 times, thus producing 500 bootstrap samples. We then examined model discrimination or concordance in each sample using the area under the curve (AUC) value. The difference between the concordance index derived from the original cohort and the average of the 500 concordance indices from the bootstrap samples was an estimate of the overfit of the prediction model. Calibration plots were also assessed across bootstrap samples. This was carried out by grouping patients into deciles based on their predicted risk and then plotting the average predicted risk against the observed risk across deciles to determine the degree of agreement.

The primary outcome and the statistical plan were pre-specified and performed as per previous studies carried out by our group [20]. All analyses were conducted using the statistical software R, version 4.2.0, and SAS version 8.3.

## 3. Results

We identified a total of 1535 TIE adults with MM who met the inclusion criteria for the study, and 1415 had baseline symptom scores available. The baseline characteristics of TIE NDMM patients are outlined in Table 1. The median age of the patients was 75, with 25.2% of the patients aged 80 years or older. With regards to previous co-morbidities, a history of congestive heart failure (13.5%) and hypertension (13.9%) were the two most commonly recorded co-morbidities. A total of 17.7% of the patients had another cancer in the 15 years preceding MM diagnosis. There were high rates of health care utilization, with 18.4% and 37.5% of patients experiencing an inpatient hospitalization or emergency department visit up to 6 months prior to the index, respectively. With regards to treatment, 84.0% of the patients had received a PI and/or IMID. With regards to functional status, the majority of patients (62.2%) had an ECOG performance status of 0–1, with 14.7% ECOG 2 and 6.8% ECOG 3–4.

Baseline symptom scores were available for 1415 patients. The four symptoms of interest, pain, tiredness, depression, and impaired well-being are found in Table 2. At baseline, 48.2% of patients reported moderate-to-severe pain, 61.8% reported moderate-to-severe tiredness, 29.3% reported moderate-to-severe depression, and 56.6% reported moderate-to-severe impaired well-being. Four distinct models were created following stepwise backward selection for the four symptoms described above. As each model was independently created, there was a slightly different set of variables that were included in each model based on the backward stepwise selection process.

For the pain model (Table 3), female sex (odds ratio (OR) 1.41) and baseline pain (OR ranging from 1.58 to 9.86 for mild-to-severe symptoms) were all associated with moderate–severe pain at one year post-index date. For tiredness (Table 3), age (≥80) (OR 1.37), baseline severe pain (OR 2.15), and baseline tiredness (OR ranging from 2.27–17.34 for mild-to-severe symptoms) were associated with moderate–severe tiredness at one year post-index date. For depression (Table 3), receiving radiation at baseline was associated with a reduced odds (OR 0.53) of experiencing future depression; however, baseline depression scores (OR ranging from 2.80 to 28.07 for mild-to-severe symptoms) were associated with moderate–severe depression one year post-index. Lastly, for impaired well-being (Table 3), age (≥80) (OR 1.48), baseline severe depression (OR 1.81), baseline severe pain (1.91), baseline tiredness (OR 2.03 and OR 2.74 for moderate and severe, respectively), and baseline impaired well-being (OR 3.78 and OR 4.12 for moderate and severe, respectively) were all associated with an increased risk of moderate–severe well-being one-year post-index.

**Figure 1:**
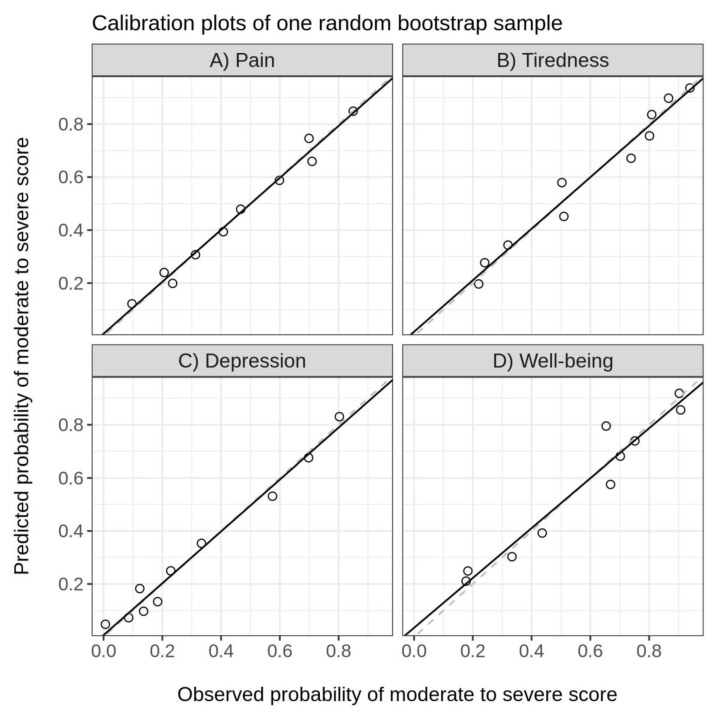
Calibration plots for symptoms: (**A**) pain, (**B**) tiredness, (**C**) depression, (**D**) well-being.

With regards to our model of fit, calibration plots were created (Figure 1). The calibration plots for pain, tiredness, depression, and well-being are good, as the points are near the 45-degree line, as desired. Overall, the model discrimination in our validation cohort was very high, with an AUC of 0.77 for pain, 0.80 for tiredness, 0.83 for depression, and 0.79 for well-being.

To highlight how these models would work, a hypothetical scenario is presented. An 81-year-old man presents with a new diagnosis of MM. He is a farmer and lives over 50 km from the nearest cancer center. On presentation, he has a hemoglobin of 82 g/L and a pathological left humerus fracture requiring radiation therapy. Prior to his diagnosis, he was active on his farm but had an ECOG of 3 at presentation. At diagnosis, he reports severe pain, moderate tiredness, moderate depression, and moderate well-being. He goes on to receive Rd therapy. The probability of him experiencing moderate-to-severe pain, tiredness, depression, or impaired well-being at 1 year from diagnosis is 70.5%, 94.3%, 83.6%, and 92.4%, respectively.

In contrast, a 75-year-old woman presents with a new diagnosis of MM. She is a retired administrative assistant and lives in a major city within a ten-minute drive to a cancer center. On presentation, her hemoglobin is 110 g/L, she is mildly symptomatic with an ECOG of 1, and has mild symptoms of pain, tiredness, depression, and impaired well-being. She goes on to receive Rd. The probability of her experiencing moderate-to-severe pain, tiredness, depression, or impaired well-being at 1 year from diagnosis is 16.1%, 13.2%, 7.6%, and 19.4%, respectively.

## 4. Discussion

### 4.1. Main Findings

Using a population cohort, we determined that the existence of pain, tiredness, depression, and impaired well-being at baseline in patients with TIE MM was predictive of these symptoms later on in their disease course, despite myeloma-directed therapy. Identifying persisting symptoms is the first step in managing these symptoms.

In our study, reporting symptoms appears to be insufficient in reducing symptoms of pain, tiredness, depression, or impaired well-being later on in their disease course. Our cohort had a high rate of health care utilization, with 18.4% and 37.5% of patients experiencing an inpatient hospitalization or emergency department visit, respectively. This likely reflects in part the poor symptom control that these patients experience. That said, we do not have data on whether interventions for moderate-to-severe symptoms were reported. In addition, the presence of pain in patients with multiple myeloma is predictive of declining health-related quality of life, independent of disease stage and clinical parameters [21]. This highlights the fact that we may be missing an important opportunity to intervene on symptoms. While the act of a patient reporting their symptoms may make a patient more self-aware, there needs to be a symptom feedback mechanism to the treating clinician. The ESAS was intended for clinician use, but in a busy oncology clinic, may be missed. One intervention could be the use of palliative care services for symptom control. We know that patients with multiple myeloma receive fewer specialist palliative care services [22], which can integrate symptom management alongside myeloma-targeted therapy. Unfortunately, even when palliative care is initiated, it is often too late in the disease trajectory [23,24]. Consideration of earlier palliative care referral for patients with reported moderate-to-severe symptoms could be considered.

We found that pain is a persisting symptom, with an OR of 9.8 of persisting one year after index date. Bone pain is one of the most common and debilitating symptoms experienced by patients with MM. The pain is due to osteolytic lesions and fractures due to increased osteoclast activity and decreased osteoblast activity. In addition, peripheral neuropathy, a frequent side effect of MM treatment, results in pain, numbness, and tingling and can severely impact function and health-related quality of life. Up to 85% of patients with MM experience bone pain at some point during their treatment trajectory [25].

Tiredness at baseline was predictive of fatigue 1 year later. Fatigue and tiredness affect 60% of patients with MM [26] and are multifactorial, with contributing factors including anemia, treatment side effects, and disease activity independent of anemia [27].

Similar to pain and tiredness, we found that depression was profoundly persistent, with an OR of 28 of moderate-to-severe depression one year following a MM diagnosis. Depression and anxiety are common in patients with MM, with a reported prevalence of 30–40%. The high prevalence is likely related to the disease itself, with its chronic and debilitating nature with persistent physical symptoms, and indefinite MM-directed therapy is associated with toxicities. General well-being was predictive of pain, tiredness, and depression at one year, likely reflecting the impact of these symptoms at baseline on well-being.

Our model utilized the ESAS, where patients report their own symptoms, in addition to clinician-reported symptoms. Patients systematically rate each symptom at each clinical encounter rather than relying on the clinician to ask about symptoms with every visit. Given that the integration of PROs into oncology care was found to improve survival and health-related quality of life and to reduce hospital resource utilization with fewer emergency room visits and hospitalizations [9], the utilization of PROs in our model helps identify vulnerable patients. PROs provide valuable insights into the effectiveness and tolerability of treatments, enabling health care providers to tailor therapies more precisely to individual needs. This proactive approach not only addresses physical symptoms but also supports emotional and psychological well-being, ultimately fostering a more comprehensive and empathetic cancer care experience.

If we can better understand the symptoms associated with MM and predict those at high risk of persisting symptoms, we can create strategies to manage these symptoms. A study of 92 patients with MM and high symptom burden participating in a myeloma-focused multidisciplinary rehabilitation program reported frequent symptoms, such as global quality of life, role functioning, fatigue, pain, peripheral neuropathy, and physical functioning [28]. The authors found that pain and fatigue were highly associated with physical functioning and health-related quality of life. Unfortunately, the primary aim of improved health-related quality of life was not met after participation in this program [28]. This study highlights the need for evidence-based guidelines for rehabilitation and palliative care for patients with MM.

For practical considerations, consider our hypothetical scenario: an 81-year-old man presents with a new diagnosis of MM. Using our model, he was identified as being at high risk for experiencing moderate-to-severe pain, tiredness, and depression or impaired well-being a year from diagnosis. Given his high risk of persistent symptoms, he may benefit from an earlier palliative care referral for symptom control.

For future work, our study highlights the need for symptom intervention management in a patient population with a high degree of symptoms. MM symptom management requires a multidisciplinary approach, including psychological support encompassing counseling and pharmacotherapy, which is crucial but is often underutilized, leading to ongoing mental health challenges. Our study and data could be utilized to generate a tool for patients, caregivers, and their providers to discuss and manage their symptoms in MM and other malignancies with high symptom burden. A future study incorporating this tool alongside an intervention such as earlier palliative care services or psycho-social support could have an impact on disease control.

### 4.2. Limitations

The data used in this study are from an Ontario, Canada, database, and therefore the results may not be generalizable to other jurisdictions with different levels of access to therapeutics. Combination therapies with PI and IMID (i.e., lenalidomide, bortezomib, and dexamethasone) or anti-CD38-based therapies were not captured in this cohort, as they were only recently approved in Ontario in this cohort; therefore, we cannot elucidate the impact of these therapies on our results. Perhaps with the improved disease control offered by combination therapies, we may have seen improved symptom control. Additionally, as this model relies on administrative databases, there are inherent limitations such as our inability to access MM disease stage, response to therapy, or treatment toxicity, which can impact prognoses. In addition, we do not have access to data on whether interventions for patient-reported moderate-to-severe symptoms were reported. The thresholds for severe symptoms are variable among individuals, and the impact of that symptom on one’s function or tolerance is better captured with health-related quality of life (HRQoL), which is not captured in ICES datasets. Prospective studies capturing HRQoL could be conducted to better understand the impact of symptoms on HRQoL. Lastly, the model considered pain, tiredness, depression, and impaired well-being as separate variables; however, they are correlated. For example, the presence of pain can have an impact on one’s mental health and therefore their depression symptoms.

## 5. Conclusions

Patients with TIE MM experience persisting symptoms of pain, tiredness, depression, and impaired well-being, despite myeloma-directed therapy. Identifying patient symptoms is the first step in developing effective management and recognizing that MM-directed therapy alone is not enough to manage the associated symptoms.

## Figures and Tables

**Table 1:** Baseline characteristics of the cohort.

Variable	Value	Total
		N = 1535
Age at index date *	Median (IQR)	75 (70–80)
	N (%) ≥ 80 years	387 (25.2)
Sex at index date	F	669 (43.6)
	M	866 (56.4)
Distance ≥ 50 km to nearest cancer center		296 (20.4)
Co-morbidities (5 years prior **)	CHF	207 (13.5)
	COPD	79 (5.2)
	Hypertension	214 (13.9)
	Diabetes	103 (6.71)
	Myocardial Infarction	106 (6.9)
Previous other cancer (15 years **)		272 (17.7)
Hemoglobin level < 100 g/L ***		490 (31.9)
Platelets < 100 billion/L		432 (28.1)
Neutrophil < 1 billion/L		143 (9.3)
Hospitalization ***		283 (18.4)
ED visit ***		576 (37.5)
Radiation ***		78 (5.1)
Novel drugs ***		1290 (84.0)
Functional score (maximum score in 6 months prior to index)	0/1	955 (62.2)
	2	226 (14.7)
	3/4	104 (6.8)
	Missing	250 (16.3)

* (One year following diagnosis.) ** Up to 3 months post-index. *** Six months prior to index; novel drugs are PI and/or IMID. IQR, interquartile range; CHF, congestive heart failure; COPD, chronic obstructive pulmonary disease; ED, emergency department.

**Table 2:** Baseline distribution of symptom scores at index date.

Baseline Symptom	Score *	Maximum Score in 6 Months Prior to Index N (%)
Pain score	0	264 (18.66)
	1–3	469 (33.14)
	4–6	395 (27.92)
	7–10	287 (20.28)
Tiredness score	0	106 (7.49)
	1–3	434 (30.67)
	4–6	474 (33.50)
	7–10	401 (28.34)
Depression score	0 *	517 (36.54)
	1–3	483 (34.13)
	4–6	260 (18.37)
	7–10	155 (10.95)
Well-being score	0	138 (9.75)
	1–3	476 (33.64)
	4–6	498 (35.19)
	7–10	303 (21.41)

* A score of 0 represents no symptoms, 1–3 mild, 4–6 moderate, and 7 or higher severe symptoms. Cohort N is 1415.

**Table 3:** Backward selection for moderate–severe pain, tiredness, depression, and well-being at one year post-index.

Variable	Value	Odds Ratio (Lower Limit–Upper Limit)			
		Pain	Tiredness	Depression	Well-Being
Baseline age at index date ≥ 80 years (REF = No)		N/A	**1.37 (1.04–1.82)**	N/A	**1.48 (1.13–1.95)**
Baseline sex at index date (REF = M)	F	**1.41 (1.12–1.77)**	N/A	N/A	1.23 (0.97–1.57)
Baseline hemoglobin level < 100 g/L (REF = no/unknown)		N/A	N/A	1.28 (0.97–1.69)	N/A
Baseline radiation (REF = No)		N/A	N/A	**0.53 (0.30–0.96)**	N/A
Baseline distance >= 50 km to nearest cancer center at index (REF = No)		0.78 (0.58–1.04)	N/A	N/A	N/A
Functional score (REF = 0/1)	2	1.08 (0.77–1.52)	1.08(0.75–1.55)	0.88 (0.61–1.28)	1.39 (0.97–2.01)
	3/4	0.80 (0.50–1.29)	1.28 (0.72–2.27)	1.46 (0.89–2.42)	1.08 (0.64–1.82)
	Missing	1.22 (0.88–1.68)	**1.56 (1.11–2.20)**	**1.50 (1.05–2.15)**	1.28 (0.92–1.78)
Baseline pain score (REF = 0)	1–3 *	**1.59 (1.09–2.31)**	0.86 (0.61–1.21)	0.94 (0.60–1.47)	0.82 (0.58–1.15)
	4–6	**5.45 (3.71–8.02)**	1.31(0.90–1.91)	1.39 (0.89–2.19)	1.37 (0.95–1.99)
	7–10	**9.86 (6.30–15.47)**	**2.15 (1.34–3.46)**	1.41 (0.86–2.31)	**1.91 (1.22–2.99)**
Baseline tiredness score (REF = 0)	1–3	1.18 (0.68–2.06)	**2.27 (1.30–3.96)**	0.67 (0.33–1.37)	1.11 (0.66–1.86)
	4–6	1.33 (0.75–2.39)	**6.56 (3.66–11.78)**	1.08 (0.52–2.22)	**2.03 (1.18–3.49)**
	7–10	1.35 (0.72–2.50)	**17.34 (9.00–33.42)**	1.14 (0.54–2.43)	**2.74 (1.52–4.95)**
Baseline depression score (REF = 0)	1–3	1.16 (0.87–1.55)	1.06 (0.80–1.42)	**2.80 (1.92–4.09)**	1.13 (0.85–1.51)
	4–6	1.23 (0.85–1.78)	1.40 (0.93–2.11)	**9.74 (6.36–14.90)**	1.16 (0.79–1.71)
	7–10	1.63 (1.01–2.64)	1.29 (0.73–2.26)	**28.07 (15.96–49.38)**	**1.81 (1.03–3.18)**
Baseline well-being score (REF = 0)	1–3	1.02 (0.62–1.68)	1.18 (0.73–1.88)	1.03 (0.51–2.05)	1.30 (0.80–2.09)
	4–6	1.64 (0.95–2.81)	1.57 (0.93–2.63)	1.24 (0.60–2.56)	**3.78 (2.25–6.35)**
	7–10	1.30 (0.72–2.35)	1.29 (0.72–2.32)	1.38 (0.64–2.98)	**4.12 (2.30–7.37)**

* Baseline scores represent the maximum score in the 6 months prior to the index date (1 year following diagnosis). N/A, not applicable. Bolded values represent *p* < 0.05.

## Data Availability

The datasets presented in this article are not readily available because the datasets from this study are held securely in coded form at ICES. While legal data-sharing agreements between ICES and data providers (e.g., health care organizations and governments) prohibit ICES from making the dataset publicly available, access may be granted to those who meet pre-specified criteria for confidential access, available at www.ices.on.ca/DAS accessed on 7 March 2024(email: das@ices.on.ca). The full dataset creation plan and underlying analytic code are available from the authors upon request, understanding that the computer programs may rely upon coding templates or macros that are unique to ICES and are therefore either inaccessible or may require modification.

## Supplementary Materials

The following supporting information can be downloaded at: https://www.mdpi.com/article/10.3390/cancers17030489/s1, Table S1. List of linked administrative databases in Ontario, Canada utilized in this project.
