# Supplementary material for: A Prognostic Symptom Model Incorporating Patient-Reported Symptoms for Transplant-Ineligible Patients with Multiple Myeloma
Source: Cancers (Basel). 2025 Feb 1;17(3):489. doi: 10.3390/cancers17030489 (PMC11817467; doi:10.3390/cancers17030489)
Supplement: Supplementary file 1 [file cancers-17-00489-s001.zip › cancers-3403611-supplementary.pdf]

Supplementary Table S1: List of linked administrative databases in Ontario, Canada utilized in this project

Database
Ontario Cancer Registry,<br>Activity Level Reporting<br>New Drug Funding Database (contains chemotherapy and radiation details), Discharge Abstract Database and National Acute Care Registry System (emergency department and hospital visit details)<br>Home Care Database<br>Registered Persons Database (RPDB)<br>Ontario Health Insurance Plan Claims Database (OHIP)<br>Ontario Drug Benefit Claims (ODB)<br>Postal Code Conversion File (PCCF)<br>Information about Ontario health care institutions funded by the Ministry of Health and Long-Term Care (INST)<br>Continuing Care Reporting System (CCRS)<br>Ontario Laboratories Information System (OLIS)<br>Ontario Asthma dataset (ASTHMA)<br>Congestive Heart Failure (CHF)<br>Ontario Hypertension dataset (HYPER)<br>Ontario Diabetes Dataset (ODD)<br>Symptom Management Database
